# Neuroendoscopic evacuation of intraventricular hematoma associated with thalamic hemorrhage to shorten the duration of external ventricular drainage

**DOI:** 10.4103/2152-7806.68342

**Published:** 2010-08-10

**Authors:** Sadahiro Nomura, Hideyuki Ishihara, Hiroshi Yoneda, Satoshi Shirao, Mizuya Shinoyama, Michiyasu Suzuki

**Affiliations:** Department of Neurosurgery, Yamaguchi University School of Medicine, Japan, Consortium of Advanced Epilepsy Treatment, Kushu Institute of Technology, Graduate School of Life Science and Systems Engineering, Japan

**Keywords:** External ventricular drainage, hydrocephalus, intraventricular hematoma, neuroendoscope, thalamic hemorrhage

## Abstract

**Background:**

We report neuroendoscopic evacuation of an intraventricular hematoma (IVH) in 13 patients with thalamic hemorrhage. We discuss strategies to improve the outcome and to shorten the management period by using external ventricular drainage (EVD).

**Methods:**

Patients were classified into fair (modified Rankin scale [mRS] grade 4 or less) and poor (mRS grade 5) outcome groups, and depending on the duration of EVD, into short (7 days or shorter) and long EVD (8 days or longer) groups.

**Results:**

The postoperative residual IVH, graded using the Graeb score, was better for the fair outcome group than for the poor outcome group (3.9 [1.2] vs. 5.7 [1.0], *P* < 0.05). The postoperative Graeb score was significantly better for the short EVD group than for the long EVD group (3.6 [0.8] vs. 6.0 [0.6], *P* < 0.01). The duration of EVD was not correlated with the IVH at the fourth ventricle, but it was correlated with the IVH at the foramen of Monro (*P* < 0.05) and the third ventricle (*P* < 0.01). Reduction in the volume of thalamic hemorrhage had no effect on the neurological outcome or duration of EVD.

**Conclusion:**

Neuroendoscopic evacuation of the IVH at the foramen of Monro and the third ventricle shortened the duration of EVD for hydrocephalus caused by thalamic hemorrhage with IVH involvement. Removal of the thalamic hemorrhage and IVH at the fourth ventricle was not necessary.

## INTRODUCTION

Thalamic hemorrhages account for 30% of all intracerebral hemorrhages (ICHs). The neurological severity of ICHs depends on the side, direction of extension, and size of the hematoma. The functional outcomes in ICHs less frequently improve by surgery than those in subcortical or putaminal hemorrhages.[9,31] Several methods of minimally invasive surgery such as stereotactic-guided evacuation[8] and its modifications[4,17,19] have been developed. Neuroendoscopic evacuation of ICHs has been the treatment modality of choice since the 1990s.[5,16,23,24]

A large thalamic hemorrhage causes obstructive hydrocephalus and involves the formation of an intraventricular hematoma (IVH). Patients with hydrocephalus require external ventricular drainage (EVD), leading to a prolonged stay in the intensive care unit, which is unfavorable from the point of view of medical economics. Neuroendoscopic surgery can be performed to treat both patients with ICH and those with IVH;[26,36] however, any further attempts at evacuation cause damage to the vessels, ependyma, and parenchyma surrounding the hematoma.

Here, we report the results of neuroendoscopic surgeries for patients with thalamic hemorrhage and IVH involvement. We analyzed the location at which the hematoma should be drained in order to improve the outcome and shorten the duration of EVD.

## MATERIALS AND METHODS

### Clinical material

Patients with obstructive hydrocephalus caused by thalamic hemorrhage with IVH involvement were included in this study (*n* = 13; five men and eight women; age range 60–77 years; average age 66.5 years). All of them had hypertensive hemorrhages, and angiography was used to rule out vascular malformations or other vasculopathies. The procedure for neuroendoscopic surgery was explained to the families of all the patients, who gave their informed consent.

### Surgical procedure and postoperative management

Surgery was performed on the day of ictus, the next day, and 2 days after the ictus in 10, 2, and 1 patient, respectively. A burr hole, ipsilateral to the thalamic hemorrhage in the frontal region, was made. Next, a ventricular tap was performed, following which a sheath was placed and a flexible neuroendoscope inserted. A surgeon positioned the tip of the endoscope on the hematoma, and an assistant evacuated the hematoma by using a 10-ml syringe connected to the irrigation channel of the endoscope. A disadvantage of the method was that nothing but the hematoma was observed when the endoscope was attached to the hematoma. In order to avoid injury to the ventricular wall, the surgeon withdrew the endoscope by a few millimeters during evacuation. Evacuation was discontinued when resistance was observed, i.e., when the endoscope touched the ventricular wall. Between the evacuations, clarity of the vision inside the ventricles was maintained by irrigation with a sufficient amount of artificial cerebrospinal fluid (CSF). Ventricular collapse during the surgery involved the risk of ventricular wall injury and was avoided by continuous irrigation. Evacuation was terminated when the aqueduct was visible. We did not insert the endoscope into the fourth ventricle or perform third ventriculostomy.

When the ICH volume was greater than 20 ml, the ICH was also evacuated after draining the IVH. By using the method described by Nishihara *et al*.,[23] we inserted a clear sheath from the same burr hole, through the same or a different tract of the ventricular tap, into the cavity of the ICH. Under observation by a rigid neuroendoscope and in a dry surgical field, the ICH was removed using a vacuum aspirator. This method for evacuating the ICH is also useful for draining the IVH when the IVH is too hard to be drained using a flexible endoscope.

EVD was performed at the end of the surgery, and intracranial pressure (ICP) was maintained at 200 mm H _2_ O by continuous CSF drainage. The EVD was discontinued using a cramp ring when the amount of CSF drained was less than 120 ml/day and no obstruction of CSF in the whole ventricles was observed on a computed tomography (CT) scan. The EVD catheter was extirpated if there was no elevation in ICP for 24 hours.

### Analysis

By using the modified Rankin scale (mRS),[33] we classified the patients into two groups on the basis of their neurological outcomes: the fair (grade 4 or lower) and poor (grade 5) outcome groups. The duration of EVD was regarded as an indicator of the period for which intensive care would be required. Depending on the duration of the EVD, the patients were divided into two groups: the short EVD (7 days or shorter) and long EVD (8 days or longer) groups. Patients needing a shunt or third ventriculostomy and patients who underwent external ventricular drain exchange because of obstruction were included in the long EVD group.

We analyzed the following factors for their association with the outcome and duration of the EVD: age, Glasgow Coma Scale (GCS) on admission, grade of thalamic hemorrhage before and after surgery, and grade of IVH before and after surgery. The severity of thalamic hemorrhage and IVH were graded according to the CT classification[18] and Graeb scale,[11] respectively. Correlation of the duration of the EVD with the location of the IVH at the body of the lateral ventricle, the foramen of Monro, and the third and fourth ventricles was also analyzed. The analyses were performed using the chi-square test and Student’s *t*-test.

## RESULTS

The GCS at admission was between 3 and 12 with an average of 7.0, which improved to 8.3 on postoperative day 3. According to the CT classification, the number of patients with grade Ib, IIb, and IIIb thalamic hemorrhage was 2, 2, and 9, respectively. In the case of four patients with ICH greater than 20 ml, evacuation was performed using the rigid endoscope and the clear sheath. In addition, ICHs in the two patients were reduced by draining into the ventricle during evacuation of the IVHs. The number of patients with grade Ib, IIb, and IIIb hemorrhage after the surgery was 5, 4, and 4, respectively. The average Graeb score was 7.5 preoperatively and 4.6 postoperatively. Three of 13 patients were operated 1 or 2 days after the ictus. We think that hardness of the IVH and difficulty of evacuation, although IVH reduction was achieved, were greater than those observed for patients operated on the day of ictus. During this study, none of the patients needed vacuum aspiration under observation with rigid endoscope because of the hardness of IVH. We attempted this method in the case of three patients and successfully reduced the IVH. No infection or other adverse events related to the surgery or EVD were recorded.

### Analysis of outcome

**Figure 1:**
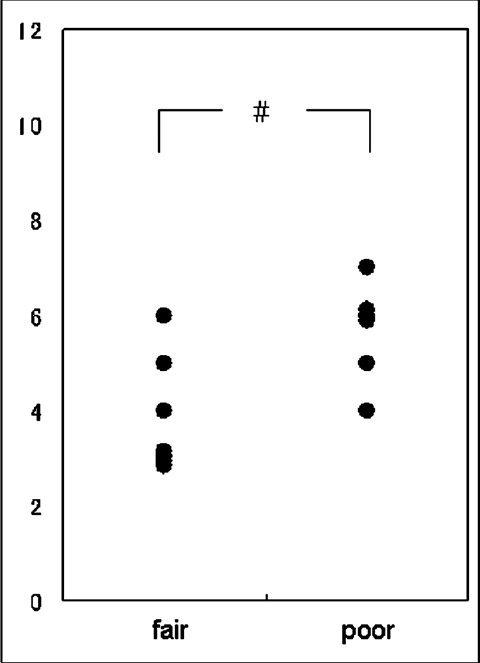
The postoperative Graeb scores for the fair outcome group and the poor outcome group. The fair outcome group has a better score than the poor outcome group (#*P* < 0.05)

The number of patients in the fair and poor outcome groups was 7 and 6, respectively. There were no symptom-free or dead patients among these 13 patients. The average age of the patients in the fair and poor outcome groups was 65.7 (9.1) years and 67.5 (7.7) years, respectively, and this difference was not significant. The average GCS on admission in the fair outcome group was 8.9 (2.5), which was higher than that in the poor outcome group (4.8 [2.6], *P* < 0.05). There was no significant difference between the pre- and postoperative CT classification of thalamic hemorrhage and in the preoperative Graeb score of the IVH. The postoperative Graeb score was better in the fair outcome group than in the poor outcome group (3.9 [1.2] vs. 5.7 [1.0], *P* < 0.05) [Figure 1].

### Analysis of the duration of external ventricular drainage

**Figure 2:**
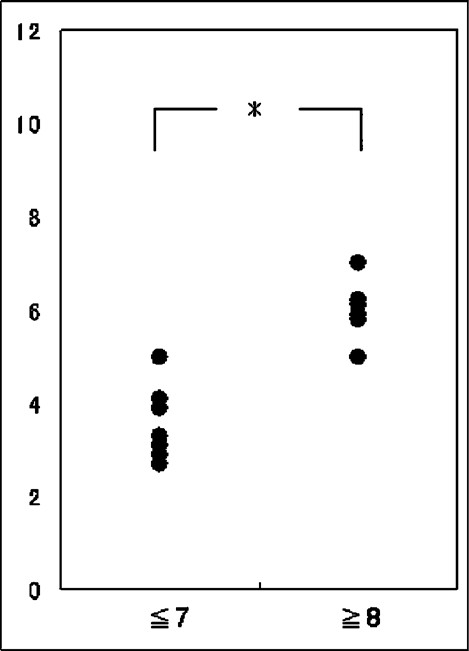
The postoperative Graeb score for the short EVD group (≤7) and the long EVD group (≥8). The short EVD group has a significantly better score than the long EVD group (*P < 0.01)

**Figure 3:**
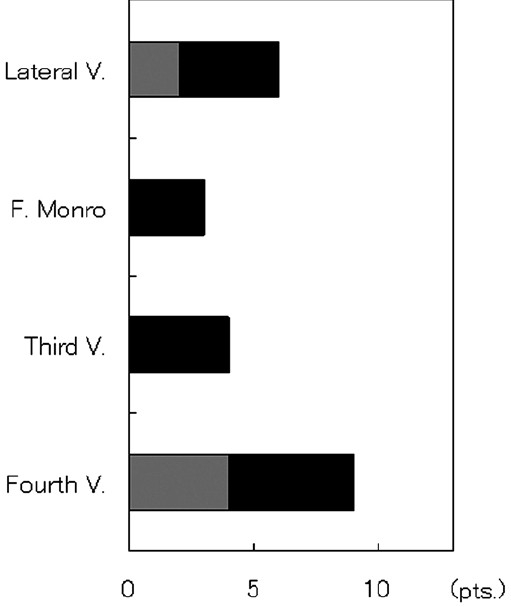
Number of patients with residual IVH at four sites after the surgery. Of the 13 patients, the IVH at the body of the lateral ventricle (Lateral V.), foramen of Monro (F. Monro), and the third (Third V.) and fourth ventricle (Fourth V.) persisted in six, three, four, and nine patients, respectively. Among these, four, three, four, and five patients, respectively, needed EVD for 8 days or longer. Light- and dark-gray columns: patients belonging to the short and long EVD groups, respectively

**Figure 4:**
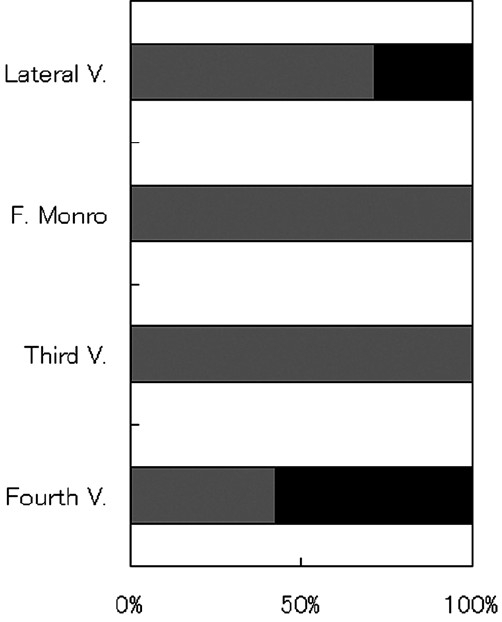
Ratio of patients with and without residual IVH after the surgery at four sites among the short EVD group. No patient exhibited residual IVH at the F. Monro or the Third V. Light- and dark-gray columns: patients belonging to the short and long EVD groups, respectively

**Figure 5:**
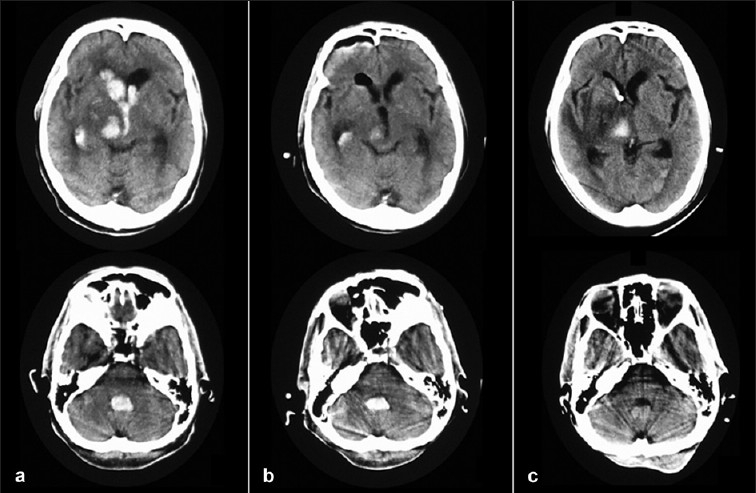
Serial CT scans of patients with thalamic hemorrhage and IVH. The IVH was observed at all the ventricles in the CT scan taken at admission (a). The IVHs at the F. Monro and the Third V. were removed, and the IVH at the fourth ventricle persisted postoperatively (b). The IVH at the fourth ventricle was not observed on the CT scan obtained on postoperative day 6 (c)

The number of patients with EVD duration of 5, 7, 12, 14, and 21 days was 2, 5, 1, 3, and 1, respectively. One patient underwent external ventricular drain exchange because of obstruction. Two patients required ventriculoperitoneal (VP) shunt or third ventriculostomy. The number of patients in the short and long EVD groups was 7 and 6, respectively. The average age of the patients in the short and long EVD groups was 69.4 [7.2] and 63.2 [8.5] years, respectively, and this difference was not significant. The GCS on admission for the patients in the short EVD group was 9.4 [2.1], which was higher than that for the patients in the long EVD group (4.2 [1.0], *P* < 0.01). There was no significant difference in the pre- and postoperative CT classification of thalamic hemorrhage and in the preoperative Graeb score of the IVH between the two groups. The postoperative Graeb score in the short EVD group was better than that in the long EVD group (3.6 [0.8] vs. 6.0 [0.6], *P* < 0.01) [Figure 2]. The removal of the IVH at the foramen of Monro and the third ventricle was easier than that at the body of the lateral ventricle or the fourth ventricle. Therefore, residual IVH at the foramen of Monro and the third ventricle was observed in three and four patients, respectively, whereas residual IVH at the body of the lateral ventricle and the fourth ventricle was found in six and nine patients, respectively. For all the four patients who had IVH at the foramen of Monro or the third ventricle, the duration of the EVD was 8 days or longer. Of the remaining patients, in the case of two of the six patients with residual IVH at the body of the lateral ventricle and in the case of four of the nine patients with residual IVH at the body of the lateral ventricle and the fourth ventricle, EVD was discontinued within 7 days [Figure 3]. On the other hand, none of the 7 patients in the short EVD group had residual IVH at either the foramen of Monro or the third ventricle after the surgery [Figure 4]. In the case of four patients with residual IVH at the fourth and without IVH at the third ventricle, the EVD was successfully discontinued within 7 days, because the IVH at the fourth ventricle lyzed spontaneously [Figure 5]. The results show that residual IVH at the foramen of Monro and the third ventricle significantly correlated with the duration of the EVD (*P* < 0.05 for the foramen of Monro and *P* < 0.01 for the third ventricle). The duration of the EVD was not correlated with IVH at the body of the lateral ventricle and the fourth ventricle.

## DISCUSSION

Although the endoscopic evacuation of ICH has been described in literature,[5,15,16,23,24] the surgical technique for removing thalamic hemorrhages has not sufficiently advanced and the indications for this technique remain controversial. On the other hand, patients with hydrocephalus caused by ventricular perforation of a thalamic hemorrhage are proven to benefit from treatment[25,26,34] because severe IVH is associated with secondary brain damage owing to increased ICP, inflammation, and edema. In addition to its pathophysiological effects in the acute stage, IVH causes communicating hydrocephalus in the chronic stage. Posthemorrhagic communicating hydrocephalus is usually ascribed to fibrosing arachnoiditis, which has been suggested to be caused by the increased deposition of extracellular matrix proteins because of the upregulation of transforming growth factor-beta (TGF-beta).[6] In order to maintain ICP, the patient’s stay in the intensive care unit is to be shortened,[13] the communicating hydrocephalus is prevented,[25] and the control of acute hydrocephalus and EVD of IVH is essential. The external ventricular drain gets easily obstructed by blood clots and carries a risk of infection. The daily infection rate of EVD increases from the time of catheter insertion, and exchanging the catheter also increases this risk.[2,14,21,32] Endoscopic surgery is beneficial for maintaining patency and allowing the termination of EVD before infection can occur.

Unlike ICH, IVH, in which the control of bleeding is difficult to achieve, should be rare because bleeding points are not located in the ventricle. During the evacuation, care should be taken not to injure the ventricular wall, choroid plexus, and veins. In particular, it should be remembered that obstruction of the veins causes edema and secondary bleeding from congestion. Further, ependymal injury disturbs the adhesion of the hematoma to the ventricular wall, in the same way that endothelial injury causes intramural thrombosis. Our results suggest that draining the IVH at the foramen of Monro and third ventricle is sufficient for continuously controlling ICP and for terminating EVD within 7 days. Yadav *et al*.[34] also emphasized the importance of clearing the third ventricle to improve CSF circulation. These results are encouraging for neuroendoscopists because they indicate that easy removal of the hematoma is effective, and it is not necessary to remove the hematoma located in a position from where its removal is difficult. A single burr hole in the frontal region enabled evacuation of the IVH, even at the contralateral foramen of Monro. On the day of ictus, the hematoma is soft and easy to evacuate even through the small lumen of the flexible endoscope. If the hematoma is too hard, it can be carefully removed using a vacuum aspirator under observation by a rigid endoscope. Water jet or ultrasound dissection[29,30] may be useful for ICH but are not suitable for IVH. Surgery should be performed before the hematoma hardens.

Some techniques for the aspiration of IVH in the fourth ventricle through the aqueduct[20] and in the contralateral lateral ventricle along with septostomy[12,20] have been previously described. Clinical trials of intraventricular fibrinolysis (IVF) with the tissue-type plasminogen activator (t-PA) for the lysis of IVH have been conducted.[10,22,27,28] It is possible to perform endoscopic third ventriculostomy during IVH removal, and this procedure has been reported to be effective[1,34] but is not always needed.[3] We believe that these procedures are not necessary, because the IVH in the fourth ventricle lyzes within a week owing to spontaneous fibrinolysis and the pulsatile movements of the CSF. CSF flow in the aqueduct is rapid and further elevated in the case of hydrocephalus.[35] This pulsatile flow is strongly correlated with ventricular morphology, especially with the total ventricular volume and third ventricle width.[7] For preventing the conduction loss of CSF pulsation to the fourth ventricle, it is important to remove the third ventricular hematoma.

Evacuation of a thalamic hemorrhage has no statistical advantage in hastening the termination of EVD. However, in individual cases, the hematoma causes a brain shift and aqueduct obstruction. In such cases, hematoma removal may contribute to reducing the resistance to CSF flow through the aqueduct.

## CONCLUSION

Neuroendoscopic evacuation of a hematoma at the foramen of Monro and third ventricle shortened the duration of EVD for hydrocephalus caused by thalamic hemorrhage. The removal of the ICH and fourth ventricular hematoma did not influence the duration of the EVD.
